# *N*-acetylcysteine add-on treatment leads to an improvement of fornix white matter integrity in early psychosis: a double-blind randomized placebo-controlled trial

**DOI:** 10.1038/s41398-018-0266-8

**Published:** 2018-10-12

**Authors:** Paul Klauser, Lijing Xin, Margot Fournier, Alessandra Griffa, Martine Cleusix, Raoul Jenni, Michel Cuenod, Rolf Gruetter, Patric Hagmann, Philippe Conus, Philipp S. Baumann, Kim Q. Do

**Affiliations:** 1 Service of General Psychiatry, Department of Psychiatry, Lausanne University Hospital (CHUV), Lausanne, Switzerland; 2 Center for Psychiatric Neuroscience, Department of Psychiatry, Lausanne University Hospital (CHUV), Lausanne, Switzerland; 3 National Center of Competence in Research (NCCR) “SYNAPSY – The Synaptic Bases of Mental Diseases”, Lausanne, Switzerland; 4 Animal Imaging and Technology Core (AIT), Center for Biomedical Imaging (CIBM), Ecole Polytechnique Fédérale de Lausanne, Lausanne, Switzerland; 5 Department of Radiology, Lausanne University Hospital (CHUV) and University of Lausanne, Lausanne, Switzerland; 6 Department of Psychiatry, Brain Center Rudolf Magnus, University Medical Center Utrecht, Utrecht University, Utrecht, Netherlands

## Abstract

Mechanism-based treatments for schizophrenia are needed, and increasing evidence suggests that oxidative stress may be a target. Previous research has shown that *N-*acetylcysteine (NAC), an antioxidant and glutathione (GSH) precursor almost devoid of side effects, improved negative symptoms, decreased the side effects of antipsychotics, and improved mismatch negativity and local neural synchronization in chronic schizophrenia. In a recent double-blind randomized placebo-controlled trial by Conus et al., early psychosis patients received NAC add-on therapy (2700 mg/day) for 6 months. Compared with placebo-treated controls, NAC patients showed significant improvements in neurocognition (processing speed) and a reduction of positive symptoms among patients with high peripheral oxidative status. NAC also led to a 23% increase in GSH levels in the medial prefrontal cortex (GSH_mPFC_) as measured by ^1^H magnetic resonance spectroscopy. A subgroup of the patients in this study were also scanned with multimodal MR imaging (spectroscopy, diffusion, and structural) at baseline (prior to NAC/placebo) and after 6 months of add-on treatment. Based on prior translational research, we hypothesized that NAC would protect white matter integrity in the fornix. A group × time interaction indicated a difference in the 6-month evolution of white matter integrity (as measured by generalized fractional anisotropy, gFA) in favor of the NAC group, which showed an 11% increase. The increase in gFA correlated with an increase in GSH_mPFC_ over the same 6-month period. In this secondary study, we suggest that NAC add-on treatment may be a safe and effective way to protect white matter integrity in early psychosis patients.

## Introduction

Mechanism-based treatments for schizophrenia are needed, given that the available treatments have limited efficacy and are often associated with serious side effects. Several lines of evidence show that redox dysregulation and oxidative stress may be a common final pathway in the pathophysiology of psychosis^1^. Abnormalities in other systems are also involved, including NMDA receptor hypofunction, neuroinflammation, and dopamine dysregulation, all of which interact in a feedforward process^2^. These mechanisms are thought to belong to a central pathophysiological hub in which an imbalance in any of these systems can lead to microscale (parvalbumin interneurons) and macroscale (white matter tracts) circuit alterations underlying disconnectivity and psychopathology^3,4^. Although some patients may lack primary redox dysregulation, any point of entry (NMDA receptor hypofunction, neuroinflammation, or dopamine dysregulation) can favor oxidative stress, which may be a common consequence of distinct etiologies (see review by Steullet et al.^2^).

Oxidative stress results from an imbalance between reactive oxygen/nitrogen species and antioxidants, resulting in macromolecular damage. Indeed, the brain is particularly vulnerable to oxidative stress given its high oxygen consumption and high content of oxidizable polyunsaturated fatty acids. Convergent evidence supports the role of oxidative stress in schizophrenia:^5^ consequences of oxidative stress, including decreased phospholipids and increased lipid peroxidation, as well as the decline of antioxidant defence systems in both the periphery and the central nervous system, were reported^6,7^. Critically, glutathione (GSH), the major non-enzymatic antioxidant and redox regulator, was decreased in the cerebrospinal fluid and prefrontal cortex in vivo^8^ and in postmortem tissue samples^9^ and was associated with negative symptoms^10^ in schizophrenia. Genetic evidence includes association with polymorphisms and copy number variations of genes related to GSH synthesis and metabolism^1,2,11,12^.

In vitro studies revealed that GSH deficit led to impairments in the proliferation and maturation of oligodendrocyte precursors^13^. Transgenic mice with a GSH deficit (*GCLM*-KO) present a decrease in mature oligodendrocytes and myelin-associated proteins in the anterior cingulate at peripuberty^13^. In another translational study from our group, we observed a decrease in fractional anisotropy in the fornix–fimbria bundle in an animal model of redox dysregulation^14^. Accordingly, we also found reduced generalized fractional anisotropy (gFA) in the fornix of early psychosis patients (EPP)^15^. Interestingly, in early psychosis, volume loss in the hippocampus correlated positively with fractional anisotropy in the fornix^15^, indicating that the integrity of these two structures is closely linked in disease. Further, smaller hippocampal volume was associated with higher blood glutathione peroxidase (GPx) activity^15^, which reflects high central oxidative status (low brain GSH), at least in male EPP^16^.

Collectively, these findings indicate that GSH and redox regulation have a central role in myelination and white matter maturation. Given that white matter alteration is a core feature of schizophrenia, these observations may lead to new, innovative treatments for use in early psychosis intervention^6,13^.

Taken together, the evidence appears to show GSH deficits and oxidative stress as promising targets in schizophrenia. Given that GSH is poorly transported across the blood-brain barrier, agents such as N-acetylcysteine (NAC) have attracted great interest as potential therapeutic tools to normalize brain GSH levels and/or redox systems. In particular, NAC, an antioxidant and precursor of GSH, is a promising candidate because it is available over the counter and almost devoid of side effects.

In a proof-of-concept clinical trial, supplementation of NAC in chronic schizophrenia patients (*N* = 140) led to improvements of negative symptoms^17^, auditory mismatch negativity^18^, and local neural synchronization in electroencephalography (EEG)^19^ as well as decreased side effects of antipsychotics^17^. Improvement in negative symptoms and total Positive and Negative Syndrome Scale (PANSS) score was replicated in three independent studies^20–22^ and two studies showed improvement in cognition in chronic schizophrenia^22,23^. Building upon this prior research, a recent double-blind randomized placebo-controlled add-on trial with NAC was carried out in EPP (*n* = 63) by Conus et al.^24^. Patients showed improved cognition (processing speed factor), and a subgroup of patients with a high baseline for peripheral oxidative status also improved in positive symptoms. However, there was no improvement in negative symptoms, potentially owing to the low rate of negative symptoms in this group. Moreover, in a subgroup of patients in the same study who underwent EEG, NAC supplementation led to improved auditory evoked potentials, known to be impaired in schizophrenia^25^.

Despite the mounting evidence that NAC may be a sustainable strategy to restore GSH deficiency and fight against oxidative stress in schizophrenia, the targets engaged in the brain by NAC have not been elucidated^26^ and have not been tested in vivo in EPP.

Recently, in vivo measurement of GSH in humans has been demonstrated on a 3-T clinical MRI scanner using short-TE ^1^H magnetic resonance spectroscopy (^1^H-MRS)^16,27^. Therefore, an essential step was achieved in the trial by Conus and colleagues^24^. In EPP who agreed to participate (*n* = 24), ^1^H-MRS was applied and revealed that NAC supplementation for 6 months actually led to an elevation of medial prefrontal GSH levels by 23%^24^.

No research has investigated whether this increase in brain GSH levels owing to NAC supplementation is accompanied by restoration/protection of white matter integrity in early psychosis^6^. Given the limited sample size of our study, we decided to focus specifically on the effect of NAC on the white matter integrity of the fornix bundle, which has been shown to be vulnerable to oxidative stress early in the time course of the illness. Moreover, we studied whether the changes in fornix integrity were linked to the changes in medial prefrontal GSH (GSH_mPFC_) levels and explained the changes in processing speed.

## Patients and methods

### Clinical trial protocol and study medication

The EPP in the present study (*N* = 20), representing a subsample of those in the original study trial^24^, have consented to be assessed with multimodal brain imaging. The flow diagram is shown in supplementary figure 1. A detailed description of the main 6-month, randomized, placebo-controlled, double-blind NAC add-on trial; the patient cohort; the study design; the sample size calculation and assessment procedures; the side effects; the efficacy; and the outcome measures has been published elsewhere^24^.

In brief, NAC (2700 mg/day) or placebo was administered to each EP patient for 6 months following a double-blinded randomized placebo-controlled design. As previously shown^17,28^, tolerability was excellent, with NAC patients showing no more side effects on the UKU scale^29^ compared with placebo^24^.

Diffusion spectrum imaging (DSI), T1 structural imaging (T1), and ^1^H-MRS were performed before NAC/placebo intake (baseline measurements) and after 6 months of NAC/placebo intake (follow-up). Symptoms were assessed with the PANSS. The processing speed factor (verbal fluency and the trail making A test, symbol coding) was extracted from the MATRICS Consensus Cognitive Battery (MCCB)^30,31^, which was administered at baseline and follow-up. Antipsychotic doses at the time of the study were converted to chlorpromazine (CPZ) equivalents in milligrams^32^ for each patient.

Following their recruitment, patients were given ID numbers, and both patients and investigators were blinded until the time of analysis, when data pooling necessitated unblinding. The hypothesis for the current study was recorded in a statistical plan prior to unblinding. The study was registered at Swiss Medic (2008DR2308) and at ClinicalTrials.gov (NCT01354132).

### Participants

The study was conducted from 2009 to 2014. All patients were recruited from TIPP (the Treatment and Early Intervention in Psychosis Program, University Hospital, Lausanne)^33^, a 3-year program specializing in the treatment of early-phase psychosis. Out of the 63 patients who participated in the original study trial, 20 patients (14 men; aged 25 ± 6.7 years) agreed to undergo DSI/T1, and 17 agreed to DSI/T1/^1^H-MRS scanning. The inclusion criteria were as follows: (1) male or female, aged 18–38 years; (2) having a psychotic disorder, defined by the “Psychosis threshold” subscale on the Comprehensive Assessment of at Risk Mental States scale (CAARMS);^34^ (3) having received under 12 months of treatment for psychosis; (4) capability to provide informed consent; (5) sufficient stability to participate in the study. The exclusion criteria were: (1) presence of clinically significant medical illnesses (including peptic ulcers), (2) organic mental disease/organic psychosis, (3) severe cerebral trauma, (4) mental retardation (intelligence quotient < 70), (5) pregnancy or lactation, (6) allergy to NAC, (7) current treatment with antioxidants, (8) poor command of French, (9) and substance-induced psychosis. All participants provided written informed consent, and the procedure was approved by the Ethics Committee of Lausanne University (10th July 2008).

NAC and placebo were kindly provided by Bioadvantex Pharma Inc. (Mississauga, Ontario, Canada) and produced under Good Manufacturing Practice conditions. All participants were randomized (by blocks of four, according to randomization lists known only to the pharmacist) in a 1:1 allocation ratio and assigned to take either effervescent NAC tablets (900 mg) at a dosage of 2700 mg/day (morning: 1800 mg; evening: 900 mg) or matching placebo tablets before meals.

### Multimodal imaging acquisition and analysis

#### gFA measured by MRI

MRI sessions were performed on a 3-Tesla scanner (Magnetom TrioTim, Siemens Medical Solutions, Erlangen, Germany) equipped with a 32-channel head coil. Each scanning session included a magnetization-prepared rapid acquisition gradient echo (MPRAGE) T1-weighted sequence with 1-mm in-plane resolution and 1.2-mm slice thickness, covering 240 × 257 × 160 voxels. The repetition (TR), echo (TE), and inversion (TI) times were 2300, 2.98, and 900 ms, respectively. The DSI sequence included 128 diffusion-weighted images with a maximum b-value of 8000 s mm^−2^ and one b0 reference image. The acquisition volume was made of 96 × 96 × 34 voxels with 2.2 × 2.2 × 3 mm resolution. TR and TE were 6800 and 144 ms, respectively.

White matter diffusion properties were estimated using gFA computed from DSI as described by Tuch^35^. Each gFA map was normalized to MNI (Montreal Neurological Institute) standard space using nonlinear registration procedures and smoothed with a Gaussian kernel of SD = 1 mm in FSL 5.0.8 (http://fsl.fmrib.ox.ac.uk/fsl/fslwiki/). Quality control included manual inspection of each gFA image for abnormalities or registration failure. The region of interest for the fornix was extracted from the JHU (Johns Hopkins University) white matter atlas^36^.

#### GSH_mPFC_ levels measured by in vivo ^1^H-MRS

The ^1^H-MRS method was described in detail in the original study trial^24^ and previous works^27,37^. In brief, the levels of GSH_mPFC_ were assessed by localized ^1^H-MRS measurements performed on a 3-T MR scanner (Magnetom TimTrio, Siemens Healthcare) with a transverse electromagnetic (TEM 3000) head coil (MR Instruments, Inc., Minneapolis, MN, USA). The magnetic field homogeneity was optimized by adjusting first- and second-order shims using FAST(EST) MAP. Single-voxel ^1^H MR spectra were acquired from a volume of interest (VOI = 20 × 20 × 25 mm^3^) in the medial prefrontal cortex using a short-TE spin-echo full-intensity acquired localized single voxel spectroscopy technique (SPECIAL) with the following scan parameters: TE/TR = 6/4000 ms, acquisition bandwidth = 2 kHz, number of averages = 148, vector size = 2048. Outer volume suppression and water suppression with variable pulse power and optimized relaxation delays were applied prior to the SPECIAL localization sequence. GSH_mPFC_ concentrations were quantified by analyzing water suppressed in vivo ^1^H MR spectra using LCModel (Stephen Provencher, Inc., Oakville, ON, Canada) with a basis set consisting of 20 simulated individual metabolite spectra and an experimentally measured macromolecule baseline. Unsuppressed water ^1^H NMR spectra were used as an internal reference. The spectral range for analysis was set to 0.2–4.2 ppm, and the Cramer-Rao bounds for GSH_mPFC_ were 10 ± 3% (mean ± s.d.).

### Statistical analysis

Statistical analyses for demographic and clinical data were performed in Prism for Mac OS X (Version 7.0c, March 1, 2017). Differences between the NAC and placebo group were assessed with *t* tests or Fisher’s exact test. A paired *t* test was used to test for interactions between time and treatment status (NAC or placebo) in each white matter voxel within the fornix. gFA was the dependent variable, whereas treatment and time were the independent variables. Age and gender were set as nuisance factors in the general linear model. Correction for multiple comparisons across all white matter voxels in the fornix was performed using a non-parametric cluster-based procedure, namely, “threshold-free cluster enhancement” with “Randomise” in FSL 5.0.8, which avoids inflation of false positives^38^. The corrected p value for the cluster was calculated from 10,000 permutations, and a *p* value < 0.05 was considered significant.

## Results

### Demographics, clinical characteristics, and longitudinal clinical changes

Among the 20 patients, 10 were part of the group that received NAC, whereas 10 received placebo. Clinical and demographic characteristics were not different between the 2 groups (Table 1). Patients treated with NAC had lower baseline GSH levels than those treated with placebo.

**Table 1:** Demographic and clinical characteristics of early psychosis patients (NAC vs placebo) at baseline (if not specified otherwise)

	NAC (*n* = 10)	Placebo (*n* = 10)	*P* value
Age (years)	25.3 ± 5.7	24.8 ± 7.9	0.3622
Gender (M/F)	9/1	5/5	0.1409
Mean CPZ, mg/day (baseline)	264.5 ± 69.29	307.8 ± 64.45	0.6531
Mean CPZ, mg/day (follow-up)	271.2 ± 65.04	418.4 ± 80.27	0.1711
PANSS baseline: positive symptoms	13.8 ± 4.8	18.0 ± 6.8	0.1249
PANSS baseline: negative symptoms	14.6 ± 4.6	18.6 ± 6.7	0.1377
PANSS baseline: total	32.6 ± 8.5	39.4 ± 9.8	0.1152
Duration of illness (days)	981.6 ± 810.2	663.9 ± 665.3	0.3743
Diagnosis			
Schizophrenia	6	6	
Schizoaffective disorder	2	1	
Bipolar disorder	1		
Major depression with psychotic features		1	
Brief psychotic episode	1	1	
Psychosis not otherwise specified		1	
Antipsychotic medication			
Quetiapine	5	3	
Clozapine	1		
Aripiprazole	1	2	
Amisulpride		2	
Risperidone	1	2	
Olanzapine	1	1	
No medication	1		
GSH_mPFC_	*n* = 9	*n* = 8	
Baseline (mM)	0.8432 ± 0.0708	1.144 ± 0.06967	0.0087
Follow-up (mM)	1.013 ± 0.08315	1.096 ± 0.07635	0.4803

*If not otherwise specified, the mean ± SD is provided. CPZ* chlorpromazine equivalents, *GSH*_*mPFC*_ glutathione concentration in the medial prefrontal cortex, *NAC* *N*-acetylcysteine, *PANSS* Positive and Negative Syndrome Scale

### Six-month longitudinal gFA changes in the fornix: NAC vs placebo

**Fig. 1:**
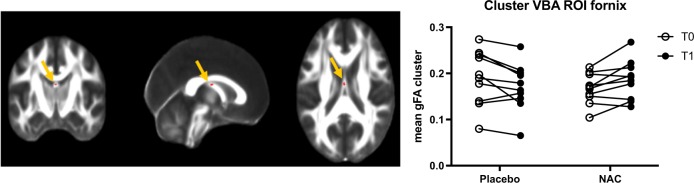
Six-month longitudinal changes in gFA: NAC vs placebo. Coronal, sagittal, and axial views (scalar gFA map) show voxel-wise analysis results indicating location (body of the fornix) of the group × time interaction, which reached significance (corrected *p* value < 0.04) (red cluster). Size of the cluster = 10 voxels. The graph shows extracted gFA values for each subject of each group (placebo, left; NAC, right) at baseline (T0) and follow-up (T1). Most placebo subjects show a decrease in gFA values, whereas NAC subjects show an increase

There was no significant difference in mean baseline gFA between NAC (0.1669 ± 0.01044) and placebo (0.1911 ± 0.01893). There was a group × time interaction, which reached significance (corrected *p* < 0.04) in the body of the fornix (size of the cluster = 10 voxels). gFA values for the subjects of each group (NAC and placebo) at baseline and follow-up are plotted (Fig. 1). Placebo subjects exhibited a decrease in mean gFA values (mean difference = − 0.02063; % change = mean difference gFA/baseline gFA × 100 = −10,795%), whereas NAC subjects showed an increase (mean difference = 0.01932; % change = + 11.576%).

### Correlation between gFA changes and GSH_mPFC_ changes over a 6-month period

**Fig. 2:**
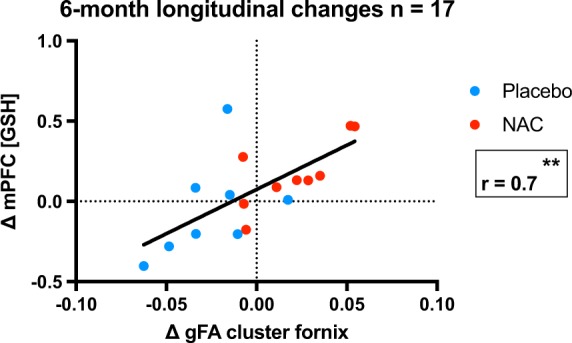
Relationship between change in gFA and change in GSH_mPFC_. Placebo patients are represented in blue, and NAC patients in red. Changes in gFA correlate with the change in GSH_mPFC_ over the 6-month period (*r* = 0.67; *p* = 0.0031)

Correlation analysis (Fig. 2) revealed a positive relationship between longitudinal change in gFA in the identified cluster in the fornix and longitudinal change in GSH_mPFC_ in the whole group (NAC and placebo; *r* = 0.67; *p* = 0.0031). When the treatment (NAC) group and the control (placebo) group were analyzed separately, the NAC group remained significant (*r* = 0.76; *p* = 0.0186), whereas the placebo group did not (*r* = 0.48; *p* = 0.2245).

When gFA was averaged over the whole fornix (supplementary figure 2), the relationship between change in gFA and change in GSH_mPFC_ in the whole sample (NAC and placebo combined) reached trend-level significance (*r* = 0.4623; *p* = 0.0617) but was nonsignificant when analyzed separately in the NAC group (*r* = 0.5254; *p* = 0.1463) and the placebo group (*r* = 0.2307; *p* = 0.5825).

There was no significant correlation between longitudinal change in average gFA extracted from the identified cluster in the fornix and longitudinal change in blood cell GPx (*r* = 0.195; *p* = 0.4101) in the whole sample (NAC and placebo combined) or in the groups analyzed separately (NAC group: *r* = 0.297; *p* = 0.4069; placebo group: *r* = 0.3476; *p* = 0.3224).

### Correlation between changes in gFA and processing speed over a 6-month period

Correlations between longitudinal change in processing speed and gFA were nonsignificant in the whole group (*r* = 0.2771; *p* = 0.2989), in the NAC group (*r* = 0.5476; *p* = 0.1710) and in the placebo group (*r* = − 0.511; *p* = 0.1956). (Supplementary figure 3).

## Discussion

We observed for the first time that the administration of NAC, a precursor of GSH, to EPP increases white matter integrity in the fornix as measured by gFA. Furthermore, longitudinal change in GSH_mPFC_ (i.e., the difference between baseline and 6 months of NAC/placebo add-on treatment) correlates with the change in gFA along the fornix bundle in NAC patients and in NAC and placebo patients pooled together. These results suggest that a GSH increase through NAC supplementation may improve/protect white matter integrity, at least in the fornix. Thus, our findings highlight that fornix integrity may improve and could represent a valid target for early psychosis intervention. The lack of significance when the whole fornix was analyzed may be linked to the decrease in sensitivity with this approach as well as the small sample size, which is the main limitation of this very demanding study for EPP in terms of scanning (multimodal MRI), design, and duration (6 months).

In the current study, we focused on the fornix bundle because its implication in schizophrenia as well as in early psychosis is well documented^15,39–41^. The fornix, as a major output of the hippocampus, has important role in cognitive processing, especially memory^41^. Notably, the hippocampus is the brain structure most robustly implicated in schizophrenia^42^. In addition, the fornix was listed among the most affected white matter tracts in a recent meta-analysis including 2000 patients with schizophrenia^43^. Progressive dysfunction in two hippocampal areas, which give rise to the fornix bundle (i.e., CA1 and subiculum), has been also very recently highlighted^44^.

Data from a *GCLM-*KO mouse model (with low GSH levels, a consequence of gene inactivation of the modulatory subunit of glutamate–cysteine ligase, the rate-limiting enzyme of GSH synthesis) was an additional strong incentive. A study by Corcoba et al.^14^ revealed a reduction in FA in the fornix–fimbria in peripubertal *GCLM-*KO mice, which remained so throughout adulthood. Furthermore, in the same KO mice, the conduction velocity of the fornix bundle was reduced in the slow-conducting fibers. This indicates that the fimbria–fornix is particularly vulnerable to GSH deficit-induced oxidative stress.

The role of redox control in white matter integrity and oligodendrocyte development has been previously documented in several ways (see review by Monin and colleagues^6^). One proposed mechanism is that redox balance regulates oligodendrocyte maturation and the switch between proliferation and differentiation^6,13,45^. Interestingly, in a mouse model, GSH deficit conditions led to impairments of proliferation and maturation of oligodendrocytes^13^.

GSH deficit is not the only mechanism that can generate oxidative stress. Indeed, it can be triggered by the perturbation of a variety of systems known to be implicated in schizophrenia, which include the redox, neuroimmune, glutamatergic, and dopaminergic systems^1,2^. As mentioned previously, these different systems do not function in isolation but interact reciprocally in a feedforward process, leading to a vicious cycle^2^. Notably, inflammatory pathways are activated by oxidative stress and vice versa^2^, and both are implicated in schizophrenia and may lead to impairment of myelination and white matter development.

NAC, as a molecule with multifaceted functions^28^, may restore or protect white matter integrity by several mechanisms. The most apparent mechanism is that NAC acts as a cysteine donor, which can be used to synthesize and replenish GSH, which, in turn, acts as a free radical scavenger. Anti-inflammatory properties have also been described for NAC, probably conferred at least in part by its antioxidant properties^2^. In preclinical models, NAC attenuates white matter injuries following a maternal immune challenge^46^. In clinical studies, NAC was added to other putative neuroprotective compounds in infantile neuronal ceroid lipofuscinosis^47^ or traumatic brain injury^48^. However, the absence of a randomized placebo-controlled design with NAC alone prevented the specific effects of NAC from being highlighted in these studies. An alternative hypothesis is that NAC may limit potential side effects of antipsychotic medication, which may impact white matter integrity^49^.

In the current study, we used a DSI sequence, characterized by strong diffusion weighting and high angular resolution. DSI is thought to be more sensitive and specific than classical diffusion tensor imaging to white matter microstructure, crossing fibers and the slow diffusion compartment^50,51^. Although it is tempting to conclude from this imaging study that NAC improves myelination, no firm conclusions can be drawn regarding the exact mechanisms. We can only speculate that “myelin maintenance and repair”^52^ is influenced by NAC. Reduction of FA may not be specific to changes in myelin content; other factors such as axonal size and coherence and changes in the volume of water spaces surrounding axons are also important^53^. Interestingly, reduction of FA may also result from inflammation^54^.

The effectiveness of NAC in early psychosis was studied by Conus et al.^24^ in the whole cohort included in the two-center trial. NAC was demonstrated to have a significant effect on neurocognition (processing speed) but not on negative symptoms. Given the efficacy of NAC in improving processing speed, we studied its relationship with gFA in the fornix; this relationship was positive, although not statistically significant.

In the study by Conus et al.^24^, subgroup exploration revealed that patients who showed improvements in their positive symptoms had higher baseline blood GPx activity than those whose positive symptoms did not improve. In other words, patients with high peripheral oxidative status benefited the most from NAC. In this context, it is interesting to note that in a previous study, high blood GPx activity was associated with small hippocampal volume^15^ and with low prefrontal GSH levels^16^. High GPx activity and/or antioxidant/redox system dysregulation may thus be a marker of response to NAC as well as a marker of small hippocampal size, which is relevant to the current study given the anatomical relationship between the fornix and the hippocampus. We thus studied the relationship between the change in GPx and the change in gFA in the fornix but did not find a significant effect. Given the small sample size, it is difficult to draw any final conclusions on this matter.

The limited sample size of the current study deserves further consideration. First, despite the absence of statistically significant difference, the two groups were not well-matched regarding sex (i.e., one female only in the NAC group) and disease severity. Second, an excess significance bias has been reported in voxel-based studies^55^ and especially in small samples^56^. This is partly owing to the wide use of parametric tests for cluster-based statistics^56^. Parametric tests rely on the assumption of a normal distribution of the data, which is often not the case when sample size is limited^57^. Here, we used non-parametric testing that does not rely on data normality and that has been shown to limit the rate of false positives in neuroimaging studies^38^. Nevertheless, these findings need to be interpreted with caution and replication in a larger randomized controlled trial is needed.

One further limitation that must be mentioned is that GSH_mPFC_ was measured in the medial prefrontal region, whereas gFA was measured in the fornix; there may be differences in GSH concentrations between brain regions. Nevertheless, we measured a longitudinal change in GSH levels, which is more likely to be proportional across brain regions. In addition, we cannot rule out the possibility that a spontaneous increase in brain GSH contributed to the observed effect in NAC-treated patients, as their basal levels were lower than those of the placebo group.

Although it is recognized that white matter anomalies are a hallmark of schizophrenia present before initiation of treatment, it has been suggested that antipsychotic medication may contribute to brain atrophy, including changes in white matter^49^. Haloperidol or olanzapine administered to macaque monkeys resulted in a tendency toward a 12.9% decrease in oligodendrocyte count^58^. In the current study, CPZ equivalents were stable in the NAC group, whereas there was a nonsignificant increase in the placebo group. Previous findings regarding the putative effects of antipsychotics on FA in patients are heterogeneous and even contradictory, with studies reporting a positive^59^, negative^60^, or null effect^61^ of antipsychotics on white matter integrity. Nevertheless, we cannot exclude that the observed effect was driven by the nonsignificant group difference in CPZ equivalents.

NAC add-on treatment is safe^24,28^ and if this preliminary study is confirmed, NAC may prove to be efficient in promoting white matter integrity in EPP, even with a mere 6 months of treatment. Further diffusion MRI studies investigating white matter changes to monitor NAC treatment response are warranted.

## Electronic supplementary material

Supplementary figure 1

Supplementary figure 2

Supplementary figure 3

Supplementary figure legends
