# Supplementary material for: N-acetylcysteine add-on treatment leads to an improvement of fornix white matter integrity in early psychosis: a double-blind randomized placebo-controlled trial
Source: Transl Psychiatry. 2018 Oct 12;8:220. doi: 10.1038/s41398-018-0266-8 (PMC6185923; doi:10.1038/s41398-018-0266-8)
Supplement: Supplementary file 1 — Supplementary figure 1 [file 41398_2018_266_MOESM1_ESM.pptx]

## Slide 1
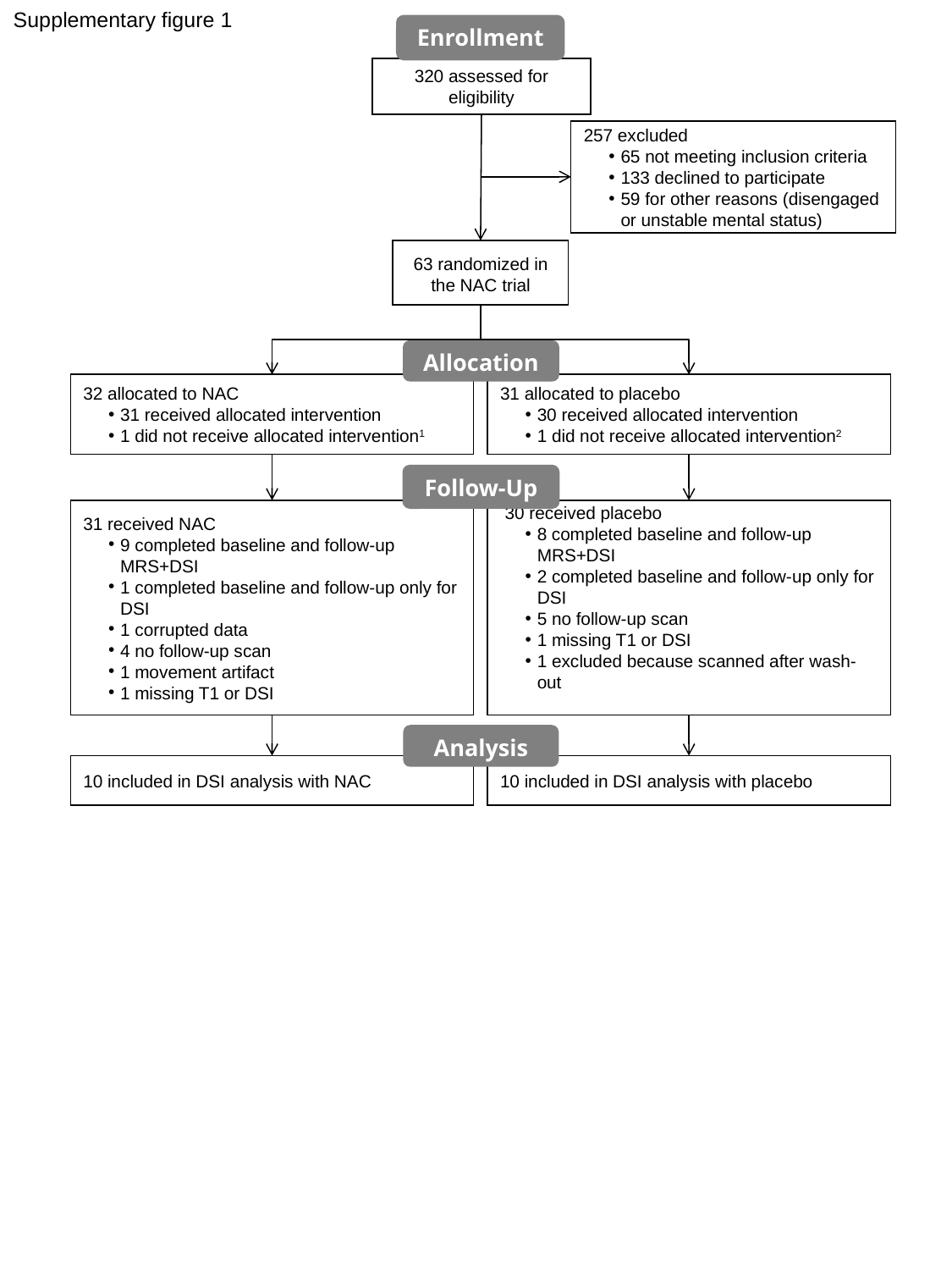

Supplementary figure 1
Enrollment
320 assessed for eligibility
257 excluded
65 not meeting inclusion criteria
133 declined to participate
59 for other reasons (disengaged or unstable mental status)
63 randomized in the NAC trial
Allocation
32 allocated to NAC
31 received allocated intervention
1 did not receive allocated intervention1
31 allocated to placebo
30 received allocated intervention
1 did not receive allocated intervention2
Follow-Up
 30 received placebo
8 completed baseline and follow-up MRS+DSI
2 completed baseline and follow-up only for DSI
5 no follow-up scan
1 missing T1 or DSI
1 excluded because scanned after wash-out
31 received NAC
9 completed baseline and follow-up MRS+DSI
1 completed baseline and follow-up only for DSI
1 corrupted data
4 no follow-up scan
1 movement artifact
1 missing T1 or DSI
Analysis
10 included in DSI analysis with NAC
10 included in DSI analysis with placebo
