# Supplementary figures and images for: N-acetylcysteine add-on treatment leads to an improvement of fornix white matter integrity in early psychosis: a double-blind randomized placebo-controlled trial
Source: Transl Psychiatry. 2018 Oct 12;8:220. doi: 10.1038/s41398-018-0266-8 (PMC6185923; doi:10.1038/s41398-018-0266-8)

## 6-month longitudinal changes n = 17

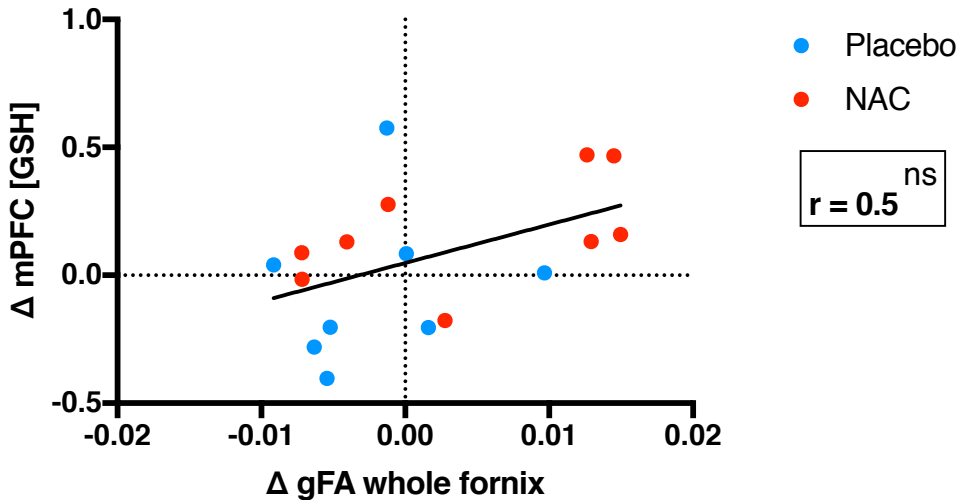

Supplement: Supplementary file 2 — Supplementary figure 2 [file 41398_2018_266_MOESM2_ESM.pdf]

## 6-month longitudinal changes n = 16

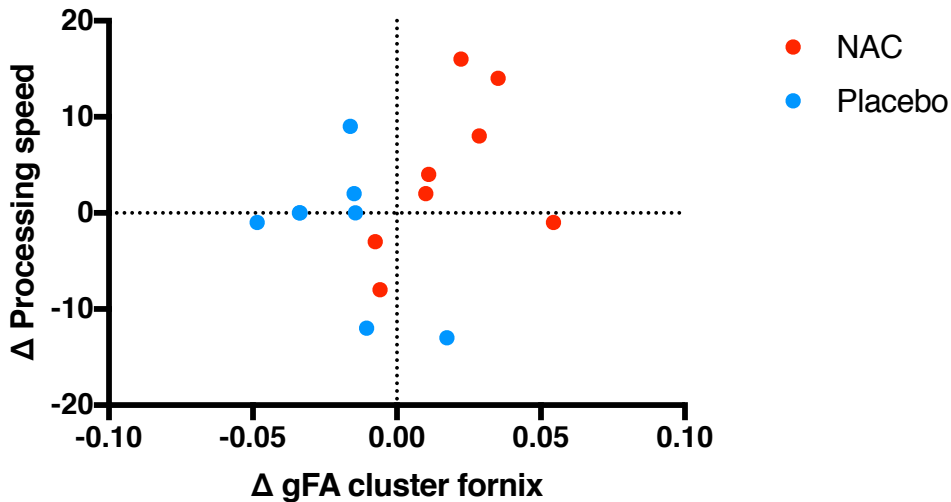

Supplement: Supplementary file 3 — Supplementary figure 3 [file 41398_2018_266_MOESM3_ESM.pdf]
