# Supplementary material for: N-acetylcysteine add-on treatment leads to an improvement of fornix white matter integrity in early psychosis: a double-blind randomized placebo-controlled trial
Source: Transl Psychiatry. 2018 Oct 12;8:220. doi: 10.1038/s41398-018-0266-8 (PMC6185923; doi:10.1038/s41398-018-0266-8)
Supplement: Supplementary file 4 — Supplementary figure legends [file 41398_2018_266_MOESM4_ESM.docx]

**Supplementary Figures legends**

**Supplementary Figure 1.** CONSORT flow diagram. Updated from Conus et al. 2018 for MRI analysis. Reasons for withdrawal from trial: ^1^ white matter lesions detected at baseline MRI scan; ^2^ withdrew at baseline.

**Supplementary Figure 2.** Correlation between change in gFA in the whole fornix and change in GSH_mPFC._ Correlation between change in gFA in the whole fornix and change in GSH_mPFC_ reached a trend level (r = 0.4623; p = 0.0617). Placebo patients are represented in blue and NAC in red.

**Supplementary Figure 3.** Relationship between change in gFA and change in processing speed. The relationship between change in gFA and processing speed was non-significant (r = 0.2771; p = 0.2989). Placebo patients are represented in blue and NAC in red.
